# Supplementary material for: Magnitude of client satisfaction and its associated factors with outpatient pharmacy service at Dubti General Hospital, Afar, North East Ethiopia: A cross sectional study
Source: PLoS One. 2021 Nov 17;16(11):e0260104. doi: 10.1371/journal.pone.0260104 (PMC8597986; doi:10.1371/journal.pone.0260104)
Supplement: S1 File — (DOCX) [file pone.0260104.s001.docx]

# S1File: Data collection tool (Questionnaire)

## Annex 1: Verbal consent form

Hello, my name is ______________________________. I am working as a data collector of the study entitled “Magnitude of client satisfaction and its associated factors with outpatient pharmacy service at Dubti General Hospital, Afar, North East Ethiopia: A cross sectional study”. The investigators of this study are Anwar Brhan Gidey, Taklo Simeneh Yazie, Tegegne Bogale, and Tesfaye Molla Gulente. I kindly request you to listen carefully about ideas that I am going to read and give me your feedback.

The objective of this study is to assess client satisfaction and associated factors at the outpatient pharmacy service. This will be helpful in improving the quality of the pharmaceutical services. You may not get direct benefit for participating in this study but your participation is valuable to identify major gaps in pharmaceutical services, which can be beneficial for the general population. It will take around 20 minutes to participate in this study. You are selected randomly and your participation is completely voluntary. You can refuse to answer any questions and/or withdraw from the study at any time without a problem to you or the services you get in the hospital. All your responses will remain strictly confidential; the hospital staff will not have access to your responses, your name will not appear on the interview guide (will not be recorded), and your responses will not be linked to your identity at any time. I assure you that your responses will be kept confidential and only used for research purpose only. For any query at any time, communicate Anwar brhan Gidey via Cell phone: 0914015492, and E-mail: anwar.brhan@gmail.com

Do you agree to participate in this study?

Yes, I agree ________

No, I don’t agree ________

# Annex 2: Questionnaire (English version interview)

**Date of Interview_____________ Time Started_____________ Time Finished ___________**

**Serial no. ____________ Signature ___________________**

**Section I. Socio-demographic Characteristics of Respondents in Dubti General Hospital**

| **No.** | **Questions** | **Response** |
| --- | --- | --- |
| 101 | Sex? | 1. Male 2. Female |
| 102 | How old are you? | __________years |
| 103 | What is your marital status? | 1. Single 2.Married 3.Divorced/Widowed |
| 104 | What is your educational level?**?** | 1. Unable to read and write  2. Able to read and write  3. Primary School (Grades 1-8)  4. Secondary (Grades 9-12)  5. Higher Education (Certificate, Diploma,  First degree & above) |
| 105 | Status of Employment? | 1.Governmental employee  2. Unemployed  3. Merchant  4. Daily laborers  5. Students  6. Pastoral |
| 106 | Where is your current residence? | 1. Rural 2. Urban |
| 107 | Frequency of Visit | 1. New visit 2. Repeat Visit |
| 108 | Service sought for during the current visit | 1. For self 2. For others |
| 109 | Payment Status |  Paying  Credit/free |
| 1010 | What is your estimated average monthly income | __________in Ethiopian birr |

**Questions on Respondents Satisfaction with pharmacy Services**

**Section II: Questions on Respondents Satisfaction with pharmacy Services**

[5 = Very satisfied (VS); 4 = Satisfied (S); 3 = Neither Satisfied nor Dissatisfied (NSD); 2 = Dissatisfied (D); 1 = Very dissatisfied (VD)]. Place **√** sign for the category you agree with most.

| **No.** | **Items** | **5** | **4** | **3** | **2** | **1** |
| --- | --- | --- | --- | --- | --- | --- |
| 1. **Structural aspect, medicine availability and cost of medication** | | | | | | |
| **I**1. | Medications I need are available. If the answer is, NO skip Q2 |  |  |  |  |  |
| **I**2. | The cost of the medication is fair |  |  |  |  |  |
| **I**3. | The pharmacy location is convenient |  |  |  |  |  |
| **I**4. | Pharmacy location related with other service is nearly located |  |  |  |  |  |
| **I**5. | The waiting area is comfortable and convenient |  |  |  |  |  |
| **I**6. | The pharmacy have organize work flow |  |  |  |  |  |
| **I**7. | The dispensary is clean |  |  |  |  |  |
| **I**8. | Dispensing area and counter convenient for service provision |  |  |  |  |  |
| 1. **Pharmacists and patient relationship** | | | | | | |
| **II**1 | The Dispenser Provide service equally to all patients |  |  |  |  |  |
| **II**2 | Dispenser is willing to answer your question? |  |  |  |  |  |
| **II**3 | The language used is easy and understandable |  |  |  |  |  |
| **II**4 | The Dispenser treat the patient with dignity and respect |  |  |  |  |  |
| **II**5 | The amount of time the pharmacist take to fill my prescription was fair |  |  |  |  |  |
| 1. **Pharmacists medication guidance** | | | | | | |
| **III**1 | The Dispenser give me medication administration instruction in understandable language |  |  |  |  |  |
| **III**2 | The Dispenser provide adequate information about medication precautions and side effects |  |  |  |  |  |
| **III**3 | The Dispenser provide adequate information about medication drug–drug and drug–food interactions |  |  |  |  |  |
| **III**4 | The Dispenser told me information about proper storage of medications |  |  |  |  |  |
| **III**5 | The Dispenser label my medicines in readable and understandable instruction |  |  |  |  |  |
| **III**6 | The counseling/advising time is sufficient |  |  |  |  |  |

**Thank you for your genuine cooperation**

**Annex 3፡ የመረጃ እና የቃል ስምምነት ቅጽ**

በሰመራ ዩኒቨርሲቲ ጤና ሳይንስ ኮሌጅ ለጥናቱ ፈቃድ ሰጭነት፤ ስለ ጥናቱ መረጃ ለተሳታፊዎች ለመስጠትና ፈቃደኝነታቸውን ለማረጋገጥ፤ ተመላላሺ ፋርማሲ ስለሚሰጠው አገልግሎት ተጠቃሚዎች ያላቸው የእርካታ መጠን መረጃ ለመሰብሰብ የተዘጋጀ መጠይቅ፣ 2012 ዓ.ም፡፡

1. **የተመላላሺ ፋርማሲ አገልግሎቱ ተጠቃሚዎች በጥናቱ ለመሳተፍ ፍቃደኝነታቸዉን የሚገልፁበት ቅጽ**

ጤና ይስጥሌኝ እኔ…… እባላለሁ፡፡ በአሁኑ ወቅት በሰመራ ዩኒቨርስቲ ጤና ሳይንስ ኮሌጅ ፍቃድ ሰጭነት በተዘጋጀው ጥናት መረጃ ሰብሳቢ ነኝ፡፡ የጥናቱ ርዕስ የተመላላሺ ፋርማሲ አገልግሎት ላይ ተጠቃሚዎች ያላቸው የእርካታ መጠን እና ተዛማጅ ጉዳዮች በዱብቲ አጠቃላይ ሆስፒታል የሚል ነው፡፡ ይህን ጥናት የሚያጠኑት አንዋር ብርሃን ግደይ፣ተገኝ ቦጋለ፣ታክሎ ስሜነህ ያዜ እና ተስፋዬ ሞላ ጉለንቴ ናቸው፡፡ በዱብቲ ሆስፒታል የአዋቂዎች ተመላላሽ መድሃኒት ክፍል የታካሚዎች እርካታ እና ተዛማጅነት ያላቸው ጉዳዮች መገምገም ዋና አላማው ሲሆን የፊርማሲ አገሌግልቱን የጥራት ደረጃ ለማሻሻል ይረዳል፣ይህንን እውን ለማድረግ በዚህ ሆስፒታል የፋርማሲ ክፍል ስላገኙት አገልግሎት ያለዎትን አስተያየት በተመለከተ የተወሰኑ ጥያቄዎችን ልጠይቅዎት እወዳለሁ፣ መጠይቁ ከጊዜዎ ከ20 ደቂቃ የማይበልጥ የሚወስድ ሲሆን በዚህ ጥናት ውስጥ የርስዎ ተሳታፊነት ሙሉ በሙሉ በርስዎ ፍቃደኝነት ላይ የተመሰረተ ነዉ፤በዚህ ጥናት ውስጥ መሳተፍዎም ሆነ ላለመሳተፍ መወሰንዎ በሆስፒታለ ውስጥ በሚያገኙት አገሌግልተ ላይ ምንም አይነት ተጽእኖ የማይኖረው ሲሆን ቃለ መጠይቁን በማንኛውም ሰአት ማቋረጥ ወይም ጥያቄዎችን አለመመለስ ይችላሉ፡፡በጥናቱ ውስጥ የተነሱት ጥያቄዎች የሚሰጡት መልስሙሉ በሙሉ በምስጢር የሚጠበቁ ሲሆን የርስዎም ስም በማንኛዉም መልኩ በጥናቱ ውስጥ አይገለጽም፡፡ በጥናቱ ለመሳተፍ ፍቃደኛ ነዎት? አዎ_________ አይደለሁም____________

ፍቃደኛ መሆናቸውን ካረጋገጡ ቃለመጠይቁን ይጀምሩ፤ፍቃደኛ ካልሆኑ ወደ ሚቀጥለው ተገልጋይ ይሸጋገሩ፡፡ ስለጥናቱ ተጨማሪ መረጃ ከፈለጉ በሚከተለው አድራሻ የጥናቱ ዋና ተመራማሪ ኣቶ ኣንዋር ብርሃን ጋር ይነጋገሩ፡፡ ስልክ ቁጥር፡0914015492፤ ኢሜል አድራሻ፡**anwar.brhan@gmail.com**

**Annex 4: ቃለ-መጠይቅ በአማርኛ**

1. **አጠቃላይ መረጃ**

**መለያ ቁጥር ______________**

**መረጃው የተሰበበት ቀን** _______**የወሰደው ጊዜ ከ______ እስከ** _____ **ፊርማ** _______

1. **የምላሽ ሰጪዎች ማህበራዊ-ኢኮኖሚያዊ ባህርያት**

| ተ.ቁ | ጥያቄዎች | መልሶች |
| --- | --- | --- |
| 101 | ፆታ | - - 1. ወንድ     2. ሴት |
| 102 | ዕድሜዎ ስንት ነው ? | __________ ዓመት |
| 103 | አሁን መኖሪያዎት የት ነው? | 1. ከተማ 2. ገጠር |
| 104 | የጋብቻ ሁኔታዎ ምንድነው? | 1. ያገባ 2. የተፋታ 3. ያላገባ |
| 105 | የትምህርት ደረጃዎ ምንድን ነው? | 1. ማንበብና መጻፍ የማይችል 2. ማንበብና መጻፍ የሚችል 3. የመጀመሪያ ደረጃ ትምህርት ቤት (1-8) 4. ሁለተኛ ደረጃ ትምህርት ቤት (9-12) 5. ከ12 በላይ (ኮሌጅ/ዩኒቨርሲቲ) |
| 106 | ሥራዎት ምንድነው? | 1. የመንግስት ሠራተኛ 2. ስራ የሌላቸው 3. ነጋዴ 4. የቀን ሰራተኛ 5. ተማሪ 6. አርብቶ አደር |
| 107 | ከዚህበፊትመጥተውያውቃሉ | 1. ለመጀመርያ ጊዜ  2. መጥቼ ኣውቃለሁ |
| 108 | መድሃኒቱን እየገዙ ያሉት ለማን ነው? | 1. ለራሴ 2. ለሌላ ሰው |
| 109 | የመድሃኒትክፍያሁኔታ? | 1.በብድር 2. በክፍያ |
| 1010 | አማካይ ወርሃዊ ገቢዎ ምን ያህል ነው ? | __________ ብር |

1. ታካሚዎች በፋርማሲ አገልግሎቱ ያላቸውን እርካታ መጠን ለማወቅ መጠይቆች

5=በጣምእስማማለሁ (በእ) 4=እስማማለሁ (እ) 3=አይታወቅም (አይ) 2=ኣልስማማም (አል) 1=በጣም ኣልስማማም (በአል)

| **ተ.ቁ** | **ጥያቄዎች** | **5** | **4** | **3** | **2** | **1** |
| --- | --- | --- | --- | --- | --- | --- |
|  | **2.1 ስለፋርማሲው ሁኔታ፤መድሃኒት አቅርቦት እና ዋጋ** | | | | | |
| 2.1.1 | የምፈልጋቸው መድሃኒቶች በፋርማሲው ላይ አሉ/አገኛለሁ |  |  |  |  |  |
| 2.1.2 | የመድሃቶች ዋጋ ተመጣጣኝ ነው |  |  |  |  |  |
| 2.1.3 | የመድሃኒት ቤቱ አቀማመጥ ለአገልግሎት አመቺ ነው |  |  |  |  |  |
| 2.1.4 | መድኃኒት ቤቱ የሚገኛበት ቦታ ከሌሎች አገልግሎቶች በቅርብ እርቀት ላይ ይገኛል? |  |  |  |  |  |
| 2.1.5 | የፋርማሲ አገልግሎት ለማግኘት የሚጠባበቁበት ቦታ ንጽህና ምቹ ነው? |  |  |  |  |  |
| 2.1.6 | የመድኃኒት አገልግሎቱ ፍሰት ጊዜ ቆጣቢ እና ምቹ ነው |  |  |  |  |  |
| 2.1.7 | መድኃኒት ቤቱ ንጽህናው የጠበቀ ነው ይስማማሉ? |  |  |  |  |  |
| 2.1.8 | የመድኃኒት ማደያ ክፍሉ እና የማስተናገጃ መስኮቶቹ ምቹ ናቸው |  |  |  |  |  |
| **2.2 ስለፋረማሲ ባለሙያዎች እና ታካሚዎች ግንኙነት** | | | | | | |
| 2.2.1 | የፋርማሲ ባለሙያ/ዋ ለሁሉም ታካሚዎች አገልግሎት በእኩል ይሰጣል/ትሰጠኛለች |  |  |  |  |  |
| 2.2.2 | የፋርማሲ ባለሙያ/ዋ ጥያቄዎች ለመመለስ ፍቃደኛ ነዉ፤ |  |  |  |  |  |
| 2.2.3 | 2የፋርማሲ ባለሙያ/ዋ የሚጠቀመው kንk ግልጽ ነበ |  |  |  |  |  |
| 2.2.4 | የፋርማሲ ባለሙያ/ዋ ታካሚዎቹን በክብር እና በአክብሮት ያስተናግዳል /ታስተናግዳለች |  |  |  |  |  |
| 2.2.5 | ባለሙያው መድሃኒቴን አዘጋጅቶ ለመስጠት (ከባሙያው መድሃኒት ለመውሰድ) የሚፈጅብኝ ጊዜ በቂ ነው |  |  |  |  |  |
| **2.3 የፋርማሲ ባለሙያዉ ታካሚዎች ስለሚወስዱት መድሃኒት የሚሰጠው ሙያዊ ምክር** | | | | | | |
| 2.3.1 | የፋርማሲ ባለሙያው /ዋ ስለ መድሀኒቴ አውሳሰድ ግልጽ በሆነ መንገድና ቋንቋ ይነግረኛል/ትነግኛለች |  |  |  |  |  |
| 2.3.2 | የፋርማሲ ባለሙያዉ/ዋ የመድሃኒቴን ቅድመ ጥንቃቄ እና  ስለ ጎንዮሽ ጉዳቶቹ በቂ መረጃ ይሰጠኛል/ትሰጠኛለች |  |  |  |  |  |
| 2.2.3 | የፋርማሲ ባለሙያው/ዋ መድሃኒቴ ከሌላ መድሃኒት ጋር እና ከምግብ ጋር ስላለው ትስስር ያስረዳኛል/ታስረዳኛለች |  |  |  |  |  |
| 2.2.4 | የፋርማሲ ባለሙያው/ዋ ስለ መድሀኒቴ ተስማሚ የአቀማመጥ ሁኔታ ይነግረኛል/ትነግኛለች |  |  |  |  |  |
| 2.2.5 | የፋርማሲ ባለሙያው /ዋ መድሃኒቶቼን በቀላሉ ሊነበብ በሚችል እና በቀላሉ ለመረዳት በሚያስችል ጽሁፍ ይጽፋል/ትጽፋለች |  |  |  |  |  |
| 2.2.6 | ለምክር አገልግሎት ባለሞያዉ የተጠቀመዉ ግዜ በቂ ነዉ |  |  |  |  |  |

**በጥናቱ ስለተሳተፉ እናመሰገናለን!!!**

**Annex 5. Questionnaire (Afarigna Version)**

**Samaran jaamiqatak ummateh barititoh.kollejik essero gacsitkee.kee.addah.oytih.cibta** Samara jaamiqat qaafiyat sayniisih kollej ummattah. qaafiyat sayniisih footima qafar agatih addal.geytima.dubtih.hospitall dawah ayfaafay geyteh tan yantifiqqime mara farmasih ayfaafayat.loonuh yaanin.mabla.keenik.gaaboysaoonuh.bicseenih.yanin essro.2012. L.GDA

- Farmas ayfaf.yantifiqqme.mari akusaaqat Qabah.agle.kee sunni.fayxi elle-yaysixxiqen cibta

Salaamaqleykum. W. W. Anu………..deqsit. Awayih uddurul. Samara jaamqatak qaafiyat saynisih.kollojik ummatah qaafiyat. saynis footimak. Kusaq buttah.adoyta kinniyo Ugutuk.yakke.kusaq.dubteh.hospital.kaxxa Mari:adadaaruk farmmasi.kak geyan footima daylima mari rufto kee.ellexayna.lehtan.caagida.fokkaqoonahn.naharsi.hadaf.kinnuk.inkih MaxxatQafiyat.nafiqik.moddaqiinoh.caddo.baxsaluk.kaaduFarmasnafqik.maxxat.caddo.yaysiisonuh.tuxiqtaTohabina.aboonuh.tahospitaalak.adawah.qarik.geyan. Ayfafal.lon.mabla.waqsiisak.uxesser.kolesseram Faxar essero.nek.beytuwayta. daguk 20 dakiik nek.beele aku saaqih. addat. kugabah.assagolli inkih.kufaxil.xissimtama Aku saaqih.addat.gaba.tasgallem kee.assagolle.waytam.madaqta.hospitaal addal geyah yan ayfaf Gibdaadinak tu alle kalah afti esero kulli waqdi. taggiriqqe aw esser Gacse waytam xiqtah. aku saaqih.addat.ugsen.esseroh.yaceen.gacsa.inkiih.qeltut.yabbi.xeeni. kinnuk. Ku mijaq. faxeem.kusaq. addat. yaysidxeenim. mafaximta.tohomay. gacisah. tan.radd. kinnan.eluk faxem.miyabbixsima

Kasaaqat tan jaluh idini inta = Yey Bale

I dinin iyyem diggowtek esser qinbisa idinin aneuleenik ciggillah yan xigoyseena fanah yabaanamaFaxe caagidil oyta geyaanan faxeenik essero esseraanam faxeenik lafale kusaf.kusaaqise cigillah yan gaubal geytaanam xiflaanah

Silk lowwo = 0914015492 kee anwar.brhan@gmail.com

Esser edde aben ayro……………

Elle qinbisen saafatay- …………… gaba kalte saafate……………..

1. **Footima inkitu;. Ayuntino qidaadoy qafiyati caalat axawah tan oytite**

| loowo | essero | Qacisa |
| --- | --- | --- |
| 101 | Nado | 1. labih 2. Sayih |
| 102 | karima magideey | __________ liqiida |
| 103 | elle yaqiise dariifa? | 1. magal 2. barri |
| 104 | Digib caalata | 1. diqibeeh  2.diqibeeh cabeeh  3.madigbinno |
| 105 | barittoh caddo macaay? | 1. Akiriy kee yakifabu maduda  2. Yakiriyeem kee yaktubbem duubat  3. 1^hyto^ caddo (1-8)  4. 2^hyto^ caddo (9-12)  5. Kolloji/jammiqata |
| 106 | Taama caalat ? | 1. doolat taama beyna  2. taama mali  3. kaloh  4 ayro taama abeyna  5. qadaaga abeyna  6. dacarsittoh xiina |
| 107 | Ahak afal teemeteh may taadigeeh | 1. nahrisi ada 2. ahak afal emeeteh adigeeh |
| 108 | Maha caagidih teemeteeh | 1.innih dawa beyah emethel  2.geerisi nummuh dawa beyah emeteeh |
| 109 | Dawa meekilah caalata | 1. tusinim 2. innih |
| 1010 | Alsi culeenti | __________birr |

1. **Daylima’mari dawa buxa nafqit loonuh yanin rufto haddo yaaxagonuh tan esseroora**

**5**. **nabam ogoolah (In.mo) 4. Ogoolah (Og.) 3. Matamixiig (ma) 2.moogola (mo) 1. inkinah moogola (nab.og)**

|  |  | In.mo | | Og. | ma | mo | Nab.og |
| --- | --- | --- | --- | --- | --- | --- | --- |
| loowo | Essero | **5** | | **4** | **3.** | **2** | **1** |
|  | - 1. **Dawa buxa ceeloo kee dawa macoy meklah ceelo macaay** | | | | | | |
| 2.1.1 | koh amirissen dawa buxah aydal yeeytohceento | |  |  |  |  |  |
| 2.1.2 | Dawa mekla fenaa fenah | |  |  |  |  |  |
| 2.1.3 | Dawah qarik mianah | |  |  |  |  |  |
| 2.1.4 | Dawa buxa elle geytimtam geeri nafqi arocak deerih xayi aracal tan | |  |  |  |  |  |
| 2.1.5 | Farmasik nafiqi geeyenoh elle qambalan afacok naddafata | |  |  |  |  |  |
| 2.1.6 | Dawa nafiqi gexsit uddur tatruse waam kee uddur bayse waa araca | |  |  |  |  |  |
| 2.1.7 | Dawa buxak nadaafatta nagay dacrisan? | |  |  |  |  |  |
| 2.1.8 | Dawa elle yacan arac kee akak yaceen boohaahi nabam meqe | |  |  |  |  |  |

| **2.2. dawa mihrat leela kee dawa beya marihan gaaraw** | | | | | | |
| --- | --- | --- | --- | --- | --- | --- |
| 2.2.1 | Dawa mihrat li/le kulle daylima marah inkih ayfaf massak yaceen |  |  |  |  |  |
| 2.2.2 | Dawa mihrat li/le essero gahsoonuh sinni fayxi loonu |  |  |  |  |  |
| 2.2.3 | Dawah mihrat li/le dawah elle beyanna aaxiqe afat yoh warseh |  |  |  |  |  |
| 2.2.4 | Dawah mihrat li/le daylima mara massakaxxa kee assakoxxal nafqi keenih yaceen |  |  |  |  |  |
| 2.2.5 | Dawah mihrat li/le dawa massossa heeh yacayuh kak beyta uddur xiqah |  |  |  |  |  |
| **2.3. Dawah mihrat li daylima mar beyahyan dawa kah yaceen mihrat kaskasoosu** | | | | | | |
| 2.3.1 | Dawah mihrat li/le dawah elle beyanna aaxiqe afat yoh warseh |  |  |  |  |  |
| 2.3.2 | Dawah mihrat li/le daalak yaakume kusaag kee gammit yan tukala duddale mascassa yoh yacee |  |  |  |  |  |
| 2.2.3 | Dawah mihrat li/le yidawa akidawallih tekkek maqollih lehtanan gaaraw yohyascassch |  |  |  |  |  |
| 2.2.4 | Dawah mihrat li/le yidawah teetih faxximta daffenah ceelo yoh warsah |  |  |  |  |  |
| 2.2.5 | Dawah mihrat li/le dawata xabcinu elle yakriyen innaakee baxcinuk elle yaaxiqenu duudan kutbe yaktuben |  |  |  |  |  |
| 2.2.6 | Elkassi macooh fafqi mihrat li eddeyintifiqe uddur xiqah |  |  |  |  |  |

- **Ne cateenimih sabbafah kulisale gadda geya**
